# Disease-specific assessment of Vision Impairment in Low Luminance in age-related macular degeneration – a MACUSTAR study report

**DOI:** 10.1136/bjophthalmol-2021-320848

**Published:** 2022-03-30

**Authors:** Jan Henrik Terheyden, Susanne G Pondorfer, Charlotte Behning, Moritz Berger, Jill Carlton, Donna Rowen, Christine Bouchet, Stephen Poor, Ulrich F O Luhmann, Sergio Leal, Frank G Holz, Thomas Butt, John E Brazier, Robert P Finger, the MACUSTAR consortium, H Agostini

**Affiliations:** 1 Department of Ophthalmology, University Hospital Bonn, Bonn, Germany; 2 Department of Medical Biometry, Informatics and Epidemiology, University Hospital Bonn, Bonn, Germany; 3 Department of Medical Biometry, Informatics and Epidemiology, University of Bonn, Bonn, Germany; 4 School of Health and Related Research, University of Sheffield, Sheffield, UK; 5 Novartis Pharma AG, Basel, Switzerland; 6 Roche Pharmaceutical Research and Early Development, Translational Medicine Ophthalmology, Roche Innovation Center Basel, Basel, Switzerland; 7 Bayer AG, Berlin, Germany; 8 UCL Institute of Ophthalmology, University College London, London, UK

**Keywords:** Diagnostic tests/Investigation, Macula

## Abstract

**Background/aims:**

To further validate the Vision Impairment in Low Luminance (VILL) questionnaire, which captures visual functioning and vision-related quality of life (VRQoL) under low luminance, low-contrast conditions relevant to age-related macular degeneration (AMD).

**Methods:**

The VILL was translated from German into English (UK), Danish, Dutch, French, Italian and Portuguese. Rasch analysis was used to assess psychometric characteristics of 716 participants (65% female, mean age 72±7 years, 82% intermediate AMD) from the baseline visit of the MACUSTAR study. In a subset of participants (n=301), test–retest reliability (intraclass correlation coefficient (ICC) and coefficient of repeatability (CoR)) and construct validity were assessed.

**Results:**

Four items were removed from the VILL with 37 items due to misfit. The resulting Vision Impairment in Low Luminance with 33 items (VILL-33) has three subscales with no disordered thresholds and no misfitting items. No differential item functioning and no multidimensionality were observed. Person reliability and person separation index were 0.91 and 3.27 for the Vision Impairment in Low Luminance Reading Subscale (VILL-R), 0.87 and 2.58 for the Vision Impairment in Low Luminance Mobility Subscale (VILL-M), and 0.78 and 1.90 for the Vision Impairment in Low Luminance Emotional Subscale (VILL-E). ICC and CoR were 0.92 and 1.9 for VILL-R, 0.93 and 1.8 for VILL-M and 0.82 and 5.0 for VILL-E. Reported VRQoL decreased with advanced AMD stage (p<0.0001) and was lower in the intermediate AMD group than in the no AMD group (p≤0.0053).

**Conclusion:**

The VILL is a psychometrically sound patient-reported outcome instrument, and the results further support its reliability and validity across all AMD stages. We recommend the shortened version of the questionnaire with three subscales (VILL-33) for future use.

**Trial registration number:**

NCT03349801.

**Key messages**

**What is already known on this topic**

- Patient relevance is key for regulatory assessment of age-related macular degeneration (AMD) treatments, but existing patient-reported outcome instruments do not fulfil development requirements by regulators or capture AMD patients’ difficulties insufficiently.

**What this study adds**

- The Vision Impairment in Low Luminance (VILL) questionnaire has been developed according to regulatory guidelines and is implemented in the MACUSTAR study. This study supports the psychometric performance including internal consistency, item fit, subscale structure, test–retest reliability and construct validity of the VILL in a multinational, multilanguage setting.

**How this study might affect research, practice or policy**

- The study supports that the VILL is sufficiently precise to capture patient-reported deficits in AMD in future trials.

## Introduction

There is a large unmet need for effective and safe treatments against onset and progression of age-related macular degeneration (AMD). However, this requires endpoints that capture disease progression reliably over the course of short interventional trials and that are accepted by regulatory authorities and health technology assessment bodies.^1–3^ Numerous structural biomarkers have previously been identified,^4–6^ but regulators agree that there is a need for patient-centred approaches, including novel functional tests and patient-reported outcomes (PROs).^3^

The visual function deficit in early and intermediate age-related macular degeneration (iAMD) is most pronounced in low-contrast and low-luminance situations, while best-corrected visual acuity under high luminance is often unaffected.^5 7–12^ Few of the available PRO instruments capture difficulties in low-luminance and low-contrast situations, which are crucial for their use as an endpoint in early and iAMD trials. The Low Luminance Questionnaire (LLQ) and the Night Vision Questionnaire (NVQ) fulfil these specific requirements but have not been developed according to regulatory guidelines, which limit their use in future interventional trials.^13–15^ Also, available instruments have not been used in the context of multinational, multilingual and multicentre studies. The Vision Impairment in Low Luminance (VILL) questionnaire, a novel vision-related quality of life (VRQoL) instrument meeting these criteria, was developed recently.^16^ In order to further assess the VILL’s psychometric performance including internal consistency, item fit, subscale structure, test–retest reliability and construct validity in a multinational/multilanguage setting, we report data from the MACUSTAR study, a European low-interventional multicentre study on iAMD progression.^1 2^

## Materials and methods

### Participants

The MACUSTAR study is a low-interventional study on the development and validation of functional, structural and patient-reported endpoints in iAMD, conducted at 20 clinical sites across Europe (Denmark, France, Germany, Italy, Netherlands, Portugal and UK).^1 2^ More details on the study’s design, assessment schedule and outcomes have been published elsewhere.^2^ In brief, an iAMD cohort (n=585) and three control cohorts (early AMD, n=34; late AMD, n=43; no AMD, n=56) were recruited. An extensive battery of functional, structural and PRO assessments (using the VILL and the generic EuroQol 5-dimension instrument, EQ-5D-5L) was performed by each participant at baseline and repeated within 2 weeks (1–3 weeks, ‘validation visit’) in a subset of 168 iAMD subjects and all subjects from the other three groups (42% of the overall sample) to assess test–retest reliability. This time frame has previously been considered appropriate to minimise recall bias.^17 18^ Disease stage was assessed independently at the test and retest visits by a central reading centre. Further visits are performed every 6 months over the entire study period for each individual but have not been included in this report. Study inclusion and disease stage classification were based on the current version of the clinical Beckman classification of AMD.^19^

The MACUSTAR study has been registered on clinicaltrials.gov.

### Vision Impairment in Low Luminance with 37 Items (VILL-37)

The VILL questionnaire was developed including in-depth interviews, focus group discussions and cognitive debriefs with patients with AMD, as outlined previously.^16^ It consists of 37 items with four response options each, plus an additional “not applicable” response option (“Didn’t do this for other reasons” / “Does not apply to me”). The VILL includes two rating scales (online supplemental table 1), referring to difficulty (items 1–24) and frequency (items 25–37). The instrument consists of the three subscales “reading and accessing information” (abbreviated reading, 20 items), “mobility and safety” (abbreviated mobility, 13 items) and “emotional well-being” (abbreviated emotional, 4 items).^16^ Within the MACUSTAR study, a PRO administration manual was provided to the study sites, ensuring similar test conditions for all participants. Questionnaires were self-administered unless participants requested interviewer administration.^2^

10.1136/bjophthalmol-2021-320848.supp1 Supplementary data

### Translation and cultural adaptation

The VILL was originally developed in Germany with German-speaking participants and subsequently translated and culturally adapted into English (United Kingdom, UK), following the principles of good practice for the translation and cultural adaptation process for PRO measures recommended by the International Society for Pharmacoeconomics and Outcomes Research (ISPOR).^20^ The English (UK) version was evaluated and optimised on the basis of clarity, grammar and spelling, uniqueness, cultural diversity and layout. Five cognitive debriefing interviews were undertaken to ensure comprehension and lack of ambiguity for each item. Inconsistencies were resolved by discussion between translators, patients and the developer (RPF). The English (UK) version then served as the source version for translation and cultural adaption of the following language versions: Danish (Denmark), Dutch (The Netherlands), French (France), Italian (Italy) and Portuguese (Portugal). Two forward translations into the target languages were provided by native speakers of the respective target language. The translations were subsequently reconciled to a single translation. The reconciled translation was then translated back into English by two independent native English speakers who were blinded to the original texts. Discrepancies were resolved in discussion. The target language versions were proofread by medical translators (native speakers of target language). Five cognitive debriefing interviews were undertaken per target language to ensure comprehension and lack of ambiguity for each item. Inconsistencies were resolved in discussion. The developer (RPF) reviewed all versions following initial translation as well as cognitive debriefs. The overall process of translation and cultural adaptation was performed in collaboration with Oxford University Innovation Ltd., following an established methodology and the ISPOR recommendations.^20–22^ All translations were undertaken by professional medical translators.

### Psychometric evaluation

Only baseline data of participants included in the study were used for analysis. Rasch analysis, derived from item response theory, was used to assess the VILL’s psychometric characteristics.^23 24^ Using the three previously established subscales of the VILL-37,^16^ a polytomous Rasch model was employed. Rasch analysis was used to assess the undimensionality of the three subscales, to identify misfitting items in each subscale, to indicate whether item levels were appropriately ordered, and to check that items did not perform differently depending on characteristics of respondents. First, a person item map was generated and relative person abilities and item difficulties were assessed. We then evaluated threshold ordering of the response categories to investigate the validity of the rating scale. Categories were collapsed where disordered thresholds were observed. To assess item misfit we considered unweighted mean square statistics. Items showing outfit or infit >mean-square value of 1.4 were removed in an initial step and item fit was re-investigated afterwards. In the case of misfitting items outside a corridor of outfit or infit mean-square values of 0.6 to 1.4, persons with misfitting responses to said item were removed and item fit was re-investigated.^25^ When this did not improve item fit, the respective item was removed. Internal consistency and the instrument’s capability of detecting different ability levels were investigated using person reliability and person separation index. Respective values above 2.0 and 0.8 were considered acceptable.^26 27^ The targeting of the instrument was assessed based on the person-item map and mean values of person measures and item measures. An absolute difference ≤1.0 logits was considered adequate.^26^ Dimensionality of the subscales was assessed based on principle component analysis (PCA) of the residuals, with a first contrast of <2.5 eigenvalues supporting unidimensionality of the subscale.^28 29^ Lastly, we investigated differential item functioning (DIF) based on gender, age group and administration mode. A significant DIF contrast ≥0.64 logits was interpreted as suggestive of biased responses in one of the analysed subgroups.^30^ P-values<0.05 were considered statistically significant.

### Statistical analysis

We performed a subgroup analysis of participants that had baseline and re-test visit data available. Person measures were obtained from Rasch analysis and statistical analysis was performed with R software V.3.6.1 (R Core Team 2020, Vienna, Austria). P-values were reported as part of the descriptive analysis and considered significant when<0.05.

#### Test–retest reliability

Intraclass correlation coefficients (ICCs) with 95% confidence intervals were calculated, accounting for repeated measures within subjects by a random effects term and interpreted following Cicchetti and Sparrow.^31^ Bland-Altman plots with limits of agreement bands were constructed and compared. Coefficients of repeatability (CoRs) were calculated as 1.96×SD of the mean differences between two measurements.^32^ Deming regression was performed and estimated intercept and slope values were compared, accounting for the variance in the test and retest datasets.^33^

#### Construct validity

The association between baseline VILL person measures and AMD disease stage was further investigated with a t-test to support construct validity of the VILL, hypothesising VILL person measures to decrease with AMD stage. To control the analysis for age, gender, the number of comorbidities and administration mode, we additionally performed linear regression analysis with the VILL person measures as dependent variables and AMD stage as an independent variable.

## Results

### Psychometric evaluation

We included 716 out of 718 MACUSTAR study participants (65% women) with baseline data in the psychometric evaluation of the VILL (table 1). Baseline data of two participants were unavailable for psychometric evaluation. Two hundred eighty-two participants were aged 55–70 years (39.4%) and 434 participants were aged 71–88 years (60.6%). All items had a low rate of not applicable or missing responses (≤20%), with the majority of these responses being not applicable to the respondent (1664 not applicable item responses; 11 missing item responses; 24 817 total valid responses). None of the items revealed floor effects, but ceiling effects (where respondents indicated no problems) were detectable in 16 items.

**Table 1:** Descriptive statistics of the overall sample and the subsample used for evaluation of repeatability

	Overall sample	Subsample
n	716	301
Age (years), mean±SD	71.9±7.0	71.2±7.2
Gender, n (%)		
Female	465 (65)	187 (62)
Male	251 (35)	114 (38)
AMD stage, n (%)		
Intermediate AMD	584 (82)*	168 (56)
No AMD	56 (8)	56 (19)
Early AMD	34 (5)	34 (11)
Late AMD	42 (6)	43 (14)
Mode of administration, n (%)		
Self-administration	191 (27)	67 (22)
Interviewer administration	525 (73)	234 (78)
Response language, n (%)		
German	241 (34)	88 (29)
English	90 (13)	27 (9)
Danish	30 (4)	12 (4)
Dutch	73 (10)	39 (13)
French	77 (11)	32 (11)
Italian	87 (12)	26 (9)
Portuguese	118 (16)	77 (26)

*Questionnaire data of two participants were not included in the overall psychometric evaluation.

AMD, age-related macular degeneration.

The four items of the emotional subscale (items 34–37) loaded positively on the first factor in the PCA of the residuals (correlation coefficient >0.4). The remaining 33 items had an unexplained variance in the first contrast of 3.49, with items related to reading / accessing information and mobility / safety forming two clusters. This confirmed the subscales previously described. As the reading and mobility subscales had an eigenvalue of the unexplained variance in the first contrast >2.0 (table 2), we re-reviewed their content, which did not reveal any further dimensions. In addition, we investigated the person measure correlation between the reading and mobility subscales and clusters of items from these subscales based on the PCA of residuals. The results did not provide evidence for multidimensionality in any of the VILL subscales (online supplemental table 2). Thus, we proceeded with the subscale structure previously identified (reading and accessing information, mobility and safety, and emotional well-being subscales).

**Table 2:** Fit parameters of the VILL-37 and VILL-33, compared with Rasch model requirements

Parameters	Rasch model	VILL-37 reading and accessing information	VILL-37 mobility and safety	VILL-37 emotional well-being	VILL-33 reading and accessing information	VILL-33 Mobility and safety	VILL-33 Emotional well-being
		20 items	13 items	4 items	17 items	12 items	4 items
Disordered thresholds	None	None	None	None	None	None	None
Misfitting items (outfit MNSQ)	0	**Item 33** (**3.29**) **Item 32** (**2.01**) **Item 29** (**1.55**)	**Item 26** (**1.46**)	None	None	None	None
Person reliability	>0.8	0.90	0.86	**0.78**	0.91	0.87	**0.78**
Person separation index	>2.0	3.07	2.52	**1.90**	3.27	2.58	**1.90**
Difference in person and item mean	<1	**1.80**	**1.95**	**1.51**	**2.09**	**2.08**	**1.51**
PCA (eigenvalue for first contrast)	<2.0	**2.37**	**2.05**	1.64	**2.34**	**2.10**	1.64
DIF: item number (DIF contrast)	<0.64						
Gender		None	None	None	None	None	None
Age group (≤70 and >70)		None	None	None	None	None	None
Administration mode*		**Item 33** (**0.81**)	None	None	None	None	None

Bold values represent misfit to the Rasch model.

*Administration mode refers to self-administration via paper forms versus interviewer administration.

DIF, differential item functioning; MNSQ, mean-square value; PCA, principal component analysis; VILL-33, Vision Impairment in Low Luminance with 33 Items; VILL-37, Vision Impairment in Low Luminance with 37 Items.

None of the category thresholds were disordered. Some of the VILL-37 items showed misfit (table 2) which was addressed by successive item reduction (see below). Two additional items of the reading subscale revealed moderate overfit before item reduction but were retained for further evaluation. Reliability indices were in an acceptable range for the reading and mobility subscales, but below the recommended thresholds for the emotional subscale (table 2). There was no evidence of multidimensionality in any subscale.

Following this, the VILL was revised based on psychometric findings. Three items from the reading subscale and one item from the mobility subscale were successively dropped due to misfit (table 2). The respective initial outfit mean-square values were 3.29, 2.01 and 1.55 for the removed reading / accessing information subscale items and 1.46 for the removed mobility / safety subscale item (online supplemental table 3). When re-investigating the psychometric properties of these two subscales after item reduction, three items of the reading subscale and one item of the mobility subscale showed initial misfit. Omitting 39 and 14 misfitting person responses to these items from the reading subscale and mobility subscales respectively, all items fit the Rasch model (online supplemental table 4). The reliability indices were in an acceptable range and no items showed DIF (table 2). The emotional subscale was less internally consistent than the reading and mobility subscales, but none of its four items showed relevant misfit or DIF (table 2). All emotional subscale items were retained. Similar to the VILL-37, person ability was higher than item difficulty in all subscales.

### Subgroup analysis

301 participants (62% women) from all study groups were included in this subgroup analysis (table 1) and complete test–retest assessments were available in 289 of these participants.

#### Test–retest reliability

**Figure 1:**
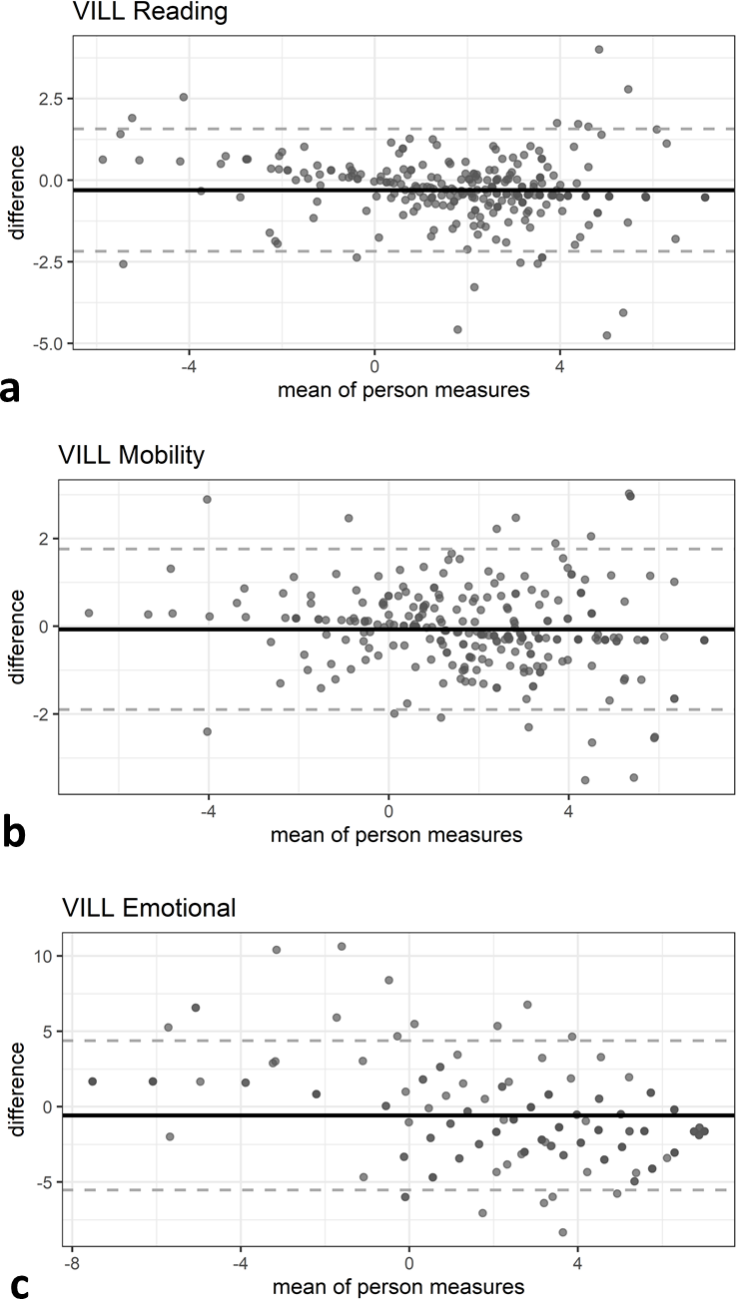
Bland-Altman plots of the VILL-33 test and retest data: (A) reading and accessing information subscale, (B) mobility and safety subscale and (C) emotional well-being subscale. AMD, age-related macular degeneration; iAMD, intermediate age-related macular degeneration; VILL-33, Vision Impairment in Low Luminance with 33 items.

ICCs of all three subscales of the VILL were excellent in the overall cohort and in the intermediate AMD subgroup (table 3). The overall ICCs of the emotional subscale were significantly lower than ICCs of the reading and mobility subscales. Mean measurement differences in Bland-Altman analysis were close to 0 (figure 1) and Deming regression supported no systematic difference between initial assessment and re-test assessment across the overall sample (table 3). However, there was a trend that persons with high person measures at baseline achieved slightly lower person measures at re-test for some of the groups (Deming regression slope <1: reading subscale: overall group; mobility subscale: overall group, early AMD, late AMD; emotional subscale: overall group, iAMD, early AMD, late AMD; table 3). Though these proportional differences were most pronounced in the emotional subscale, they were not observed for the reading or mobility subscale in participants with iAMD.

**Table 3:** Test–retest reliability statistics of the VILL-33 subscales per AMD stage

		Overall	iAMD	No AMD	Early AMD	Late AMD
		n=289	n=167	n=53	n=28	n=41
VILL reading and accessing information	ICC (95% CI)	0.920 (0.900 to 0.936)	0.868 (0.825 to 0.901)	0.736 (0.584 to 0.838)	0.958 (0.912 to 0.980)	0.878 (0.783 to 0.933)
	CoR	1.9	1.7	2.4	0.5	2.2
	Deming intercept (95% CI)	−0.14 (−0.29 to 0.01)	−0.18 (−0.37 to 0.01)	−0.23 (−0.82 to 0.37)	−0.25 (−0.57 to 0.07)	−0.22 (−0.65 to 0.21)
	Deming slope (95% CI)	0.93 (0.87 to 0.98)*	0.93 (0.85 to 1.01)	0.97 (0.77 to 1.18)	0.95 (0.89 to 1.02)	0.87 (0.68 to 1.06)
VILL mobility and safety	ICC (95% CI)	0.929 (0.911 to 0.943)	0.904 (0.872 to 0.928)	0.800 (0.677 to 0.879)	0.974 (0.944 to 0.988)	0.932 (0.876 to 0.963)
	CoR	1.8	1.7	2.4	0.9	1.7
	Deming intercept (95% CI)	0.04 (−0.08 to 0.17)	−0.10 (−0.31 to 0.11)	0.07 (−0.52 to 0.66)	0.19 (−0.15 to 0.53)	0.05 (−0.21 to 0.30)
	Deming slope (95% CI)	0.94 (0.89 to 0.99)*	0.97 (0.88 to 1.06)	0.99 (0.75 to 1.23)	0.90 (0.82 to 0.97)*	0.88 (0.78 to 0.97)*
VILL emotional well-being	ICC (95% CI)	0.822 (0.78 to 0.856)	0.791 (0.727 to 0.842)	0.632 (0.44 to 0.77)	0.895 (0.788 to 0.95)	0.769 (0.608 to 0.869)
	CoR	5.0	4.5	4.4	3.6	6.3
	Deming intercept (95% CI)	0.06 (−0.19 to 0.31)	0.14 (−0.20 to 0.47)	−1.47 (−3.78 to 0.83)	0.03 (−0.47 to 0.52)	0.27 (−0.52 to 1.06)
	Deming slope (95% CI)	0.71 (0.67 to 0.75)*	0.69 (0.63 to 0.75)*	0.95 (0.60 to 1.30)	0.75 (0.68 to 0.81)*	0.70 (0.60 to 0.80)*

*CIs excluding the expected intercept 0 (systematic difference between the two measurements) or the expected slope 1 (disproportionate slope) were marked.

AMD, age-related macular degeneration; CoR, coefficient of repeatability; iAMD, intermediate age-related macular degeneration; ICC, intraclass correlation coefficient; VILL-33, Vision Impairment in Low Luminance with 33 Items; VILL, Vision Impairment in Low Luminance.

#### Construct validity

**Figure 2:**
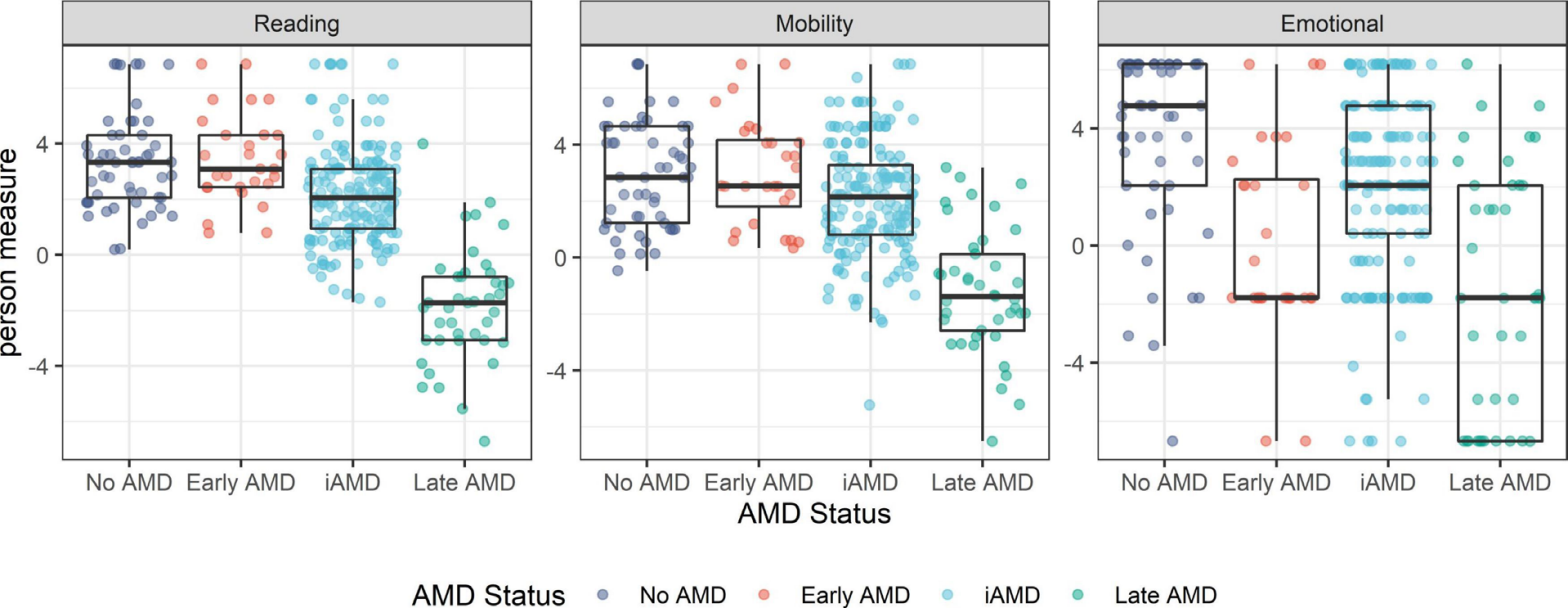
Distributions of VILL-33 person measures across different age-related macular degeneration stages: (A) reading and accessing information subscale, (B) mobility and safety subscale and (C) emotional well-being subscale. VILL, Vision Impairment in Low Luminance; VILL-33, Vision Impairment in Low Luminance with 33 items.

The mean person measures of all subscales of the VILL differed noticeably between AMD stages (figure 2). Higher person measures indicate better VRQoL. Mean person measures were significantly lower in the late AMD group than in the iAMD group (p<0.0001 for all three subscales). Person measures of all three VILL subscales were significantly lower in the iAMD group than in the no AMD group (p<0.0001, reading; p=0.0053, mobility; p=0.0011, emotional). Person measures of the reading and mobility subscale were significantly lower in the iAMD group than in the early AMD group (p=0.0006, reading; p=0.0197, mobility). This did not apply to the emotional subscale (early AMD<iAMD person measures, p=0.01). In linear regression analysis, all VILL subscale person measures were significantly associated with late AMD (p<0.0001) when controlling for age, gender, number of comorbidities and mode of administration. In addition, the reading and emotional subscale person measures were associated with iAMD (p=0.001 and 0.0003, respectively) and the emotional subscale person measures were associated with early AMD (p<0.0001).

## Discussion

The VILL is a novel PRO instrument developed to meet the regulatory requirements for use in AMD trials, with a focus on intermediate AMD. Based on this further evaluation in the MACUSTAR study, we recommend the use of the 33-item VILL with its three subscales reading / accessing information, mobility / safety and emotional well-being. The Vision Impairment in Low Luminance with 33 items (VILL-33) has good psychometric properties, high test–retest reliability and adequate construct validity.

The VILL-37 questionnaire was developed according to regulatory standards.^16^ Using data from the MACUSTAR study, we have continued an ongoing validation process following regulatory guidelines to be able to support labelling claims in the context of future drug trials.^34^ Overall, MACUSTAR participants were on average younger (mean age 72±7 years) than the cohort in which the VILL was developed (mean age 76±7 years). Noticeably, a lower proportion in the MACUSTAR cohort had late AMD (6% in the MACUSTAR sample, 42% in the development study).^16^ Both the initial development study and the present study are supportive of the internal consistency of the reading and mobility subscales of the VILL with person reliability and person separation values within the accepted ranges. Unlike the VILL-37, no items of the VILL-33 showed misfit.

The emotional subscale had a lower internal consistency than the reading and mobility subscales in the MACUSTAR data which is similar to the development study. Also, repeatability and construct validity were worse for the emotional subscale than for the other subscales of the VILL. These findings may be related to the lower number of items in the emotional subscale (four items) than in the reading (17) or mobility subscales (12 items) which could make the subscale more prone to measurement noise. The broad definition of the construct “emotional well-being” in the VILL, which was based on experiences of AMD patients and content from existing PRO instruments but not specifically obtained or validated in the context of psychiatric comorbidities may also explain why the emotional subscale appears to be less reliable and construct valid than the reading and mobility subscales. However, we retained the emotional subscale on the basis of content validity while acknowledging the need to explore reliability and validity of this subscale further, including an exploration of its concurrent validity in the context of existing instruments measuring the underlying psychological concepts including worry, anxiety and depression.

We recommend the VILL-33 to be used in future applications over the VILL-37. Both the VILL-33 and the VILL-37 were not well targeted to the MACUSTAR study sample, and ceiling effects were more prominent in the MACUSTAR data than in the VILL development study.^16^ This is likely due to the very good vision of the large majority of MACUSTAR participants at baseline who report greater ability than that required to perform several of the items. However, as the VILL was developed to capture changes in VRQoL associated with disease progression within iAMD and to late AMD, and has been shown to be appropriate for a sample with a larger proportion of late AMD participants, we are confident it will perform adequately in the longitudinal part of the MACUSTAR study as it retains scope to capture reduction in VRQoL as progression ensues. Against this background, several items were retained despite ceiling effects.

Besides the VILL, only a limited number of PRO instruments were designed to capture the characteristic impairment of patients with AMD under low-luminance and low-contrast conditions, that is, the LLQ and the NVQ. The LLQ was designed based on focus group discussions with 80 patients with AMD and patients with inherited retinal disease and was administered to 125 participants including individuals with normal ageing changes.^8^ In psychometric testing using classical test theory, ceiling effects were present in a high proportion of items; for example, in 22% of the items obtained, the full sum score in all items related to general dim lighting problems.^8^ The validated German version of the LLQ included 23 of the 32 original items and was evaluated using a Rasch model in 274 participants (including 90 controls).^35^ While the instrument showed good internal consistency, item targeting was poor due to ceiling effects (difference in person and item mean 2.1). Though the targeting parameter in our study was similar, our population is not directly comparable to the population from the German LLQ validation study.^35^ Test–retest reliability of the reading and mobility VILL subscales was higher and the sample size larger than the available repeatability data of the LLQ-32 (Pearson correlation coefficents 0.46–0.88 in 60 participants).^8^ ICC and CoR values of the VILL were also similar to the Vision and Night Driving Questionnaire, which is specifically targeted at an elderly, driving population with good visual function.^36^

Validation of the NVQ was originally based on 1052 participants of the Complications of AMD Prevention Trial.^37^ Again, internal consistency was good, but the instrument suffered from ceiling effects. A recent study investigated NVQ-10 responses of participants of the Laser Intervention in Early Stages of Age-Related Macular Degeneration study.^14 38^ Rasch analysis revealed disordered thresholds, poor discriminatory power of the items and underfit of items, as well as poor person separation (internal consistency). The authors recommended the NVQ-10 not to be used in iAMD samples based on these findings. Unlike the NVQ, the psychometric analysis of the VILL revealed good internal consistency, item fit and functioning of the rating scale, supporting use of the VILL in future AMD studies.

A key strength of our study is its large, well-phenotyped sample, including confirmation of AMD staging by a central reading centre as well as central and on-site monitoring to ensure the study meets high quality requirements. Use of the current reference standard of item response theory enabled us to evaluate the VILL at quality standards that cannot be reached using classical test theory.^39^ However, despite its large overall sample, we did not evaluate differential item functioning between different language versions which needs to be examined in future studies.^40^ We have neither included functional data of the participants in our analyses nor investigated structural biomarkers besides AMD stage as both aspects were beyond the scope of this paper. The study groups (iAMD group and control groups) were not balanced in terms of age or participant characteristics, which may have affected the comparisons between disease stages.

To conclude, we provide additional evidence for the validity of the VILL questionnaire in AMD based on MACUSTAR data. We recommend the shortened version of the questionnaire with 33 items (VILL-33) for use in future studies.

## Data Availability

Data are available upon reasonable request. The datasets used in the present study are available from the MACUSTAR consortium upon reasonable request.
