# Supplementary material for: Disease-specific assessment of Vision Impairment in Low Luminance in age-related macular degeneration – a MACUSTAR study report
Source: Br J Ophthalmol. 2022 Mar 30;107(8):1144–50. doi: 10.1136/bjophthalmol-2021-320848 (PMC10359508; doi:10.1136/bjophthalmol-2021-320848)
Supplement: Supplementary data [file bjophthalmol-2021-320848supp001.pdf]

Supplementary Table 1: VILL item set (English, UK version)

Item	Rating scale	Subscale	VILL-37	VILL-33
Adjusting to the dark when entering a dimly lit room? (e.g. a restaurant at night)	Difficulty	Reading and accessing information	●	●
Recognizing small objects in dim lighting? (e.g. coins)	Difficulty	Reading and accessing information	●	●
Recognizing people's faces outside during dusk?	Difficulty	Reading and accessing information	●	●
Recognizing people or objects by candlelight?	Difficulty	Reading and accessing information	●	●
Seeing things clearly close up in the middle of your field of vision?	Difficulty	Reading and accessing information	●	●
Reading print which has a low contrast to its background?	Difficulty	Reading and accessing information	●	●
Reading print which is not black? (e.g. grey)	Difficulty	Reading and accessing information	●	●
Reading text on a digital display? (e.g. in the car, on an electronic radio)	Difficulty	Reading and accessing information	●	●
Reading print against a colourful background? (e.g. a brochure)	Difficulty	Reading and accessing information	●	●
Reading a paperback novel in dim lighting?	Difficulty	Reading and accessing information	●	●
Reading a newspaper in dim lighting?	Difficulty	Reading and accessing information	●	●
Reading a menu in a dimly lit restaurant?	Difficulty	Reading and accessing information	●	●
Reading labels or instructions on medicine bottles in good lighting?	Difficulty	Reading and accessing information	●	●
Reading labels or instructions on medicine bottles in dim lighting?	Difficulty	Reading and accessing information	●	●
Reading package labels or price tags in a shop?	Difficulty	Reading and accessing information	●	●
Driving a car on a sunny day? (with or without sunglasses)	Difficulty	Mobility and safety	●	●
Driving a car along a road lined with trees on a sunny day? (with or without sunglasses)	Difficulty	Mobility and safety	●	●
Driving a car at night?	Difficulty	Mobility and safety	●	●
Driving a car at night in the rain?	Difficulty	Mobility and safety	●	●
Reading street signs in time when driving by?	Difficulty	Mobility and safety	●	●
Walking on uneven ground in the dark?	Difficulty	Mobility and safety	●	●
Going out to do things during dusk? (e.g. visiting the supermarket or shops)	Difficulty	Mobility and safety	●	●
Seeing steps or curbs in the dark?	Difficulty	Mobility and safety	●	●
Getting your bearings in dimly lit or dark unfamiliar places?	Difficulty	Mobility and safety	●	●
Felt blinded by oncoming cars at night?	Frequency	Mobility and safety	●	●
Noticed lights to be blurred or fuzzy at night? (e.g. street lights)	Frequency	Mobility and safety	●	
Felt blinded by the sun whilst driving a car? (with or without sunglasses)	Frequency	Mobility and safety	●	●
Felt unsafe as a pedestrian or cyclist at dawn or at night?	Frequency	Mobility and safety	●	●
Felt blinded whilst reading in the sun?	Frequency	Reading and accessing information	●	
Felt exhausted by reading in dim light?	Frequency	Reading and accessing information	●	●
Needed additional lighting to see or read anything?	Frequency	Reading and accessing information	●	●
Used magnifiers or other visual aids?	Frequency	Reading and accessing information	●	

Terheyden JH, *et al. Br J Ophthalmol* 2023; 107:1144–1150. doi: 10.1136/bjophthalmol-2021-320848

Used contrast enhancing tints?	Frequency	Reading and accessing information	●	
Felt worried that your eyesight might get worse?	Frequency	Emotional well-being	●	●
Felt worried about losing your independence?	Frequency	Emotional well-being	●	●
Felt worried about the future?	Frequency	Emotional well-being	●	●
Felt worried that your lifestyle might change due to your eye condition?	Frequency	Emotional well-being	●	●

Supplementary Table 2: Person measure correlation between the reading and accessing information and mobility and safety subscales and clusters of items from these subscales

Subscale	Cluster*	Items	Pearson-r#
Reading and accessing information	1	10, 11, 12	0.781
	3	1, 2, 3, 4, 5, 29, 30, 31, 32, 33	0.928
Mobility and safety	1	21, 23, 24	0.794
	3	16, 17, 18, 19, 25	0.874

*based on a principal component analysis of residuals
#correlation with person measures from the VILL-37 subscale

Supplementary Table 3: Misfitting VILL-37 items and steps of item reduction

Subscale	Item reduction step	Misfitting items	Infit mean-square	Outfit mean-square
Reading and accessing information	0	33	2.75	3.29
	0	32	1.74	2.01
	0	29	1.30	1.55
	0	10	0.61	0.59
	0	11	0.61	0.59
	1*	32	1.89	2.20
	1*	29	1.40	1.68
	2#	29	1.53	1.83
	3¶	none	n/a	n/a
Mobility and safety	0	26	1.59	1.46
	1§	none	n/a	n/a

*item 33 dropped, #item 32 dropped, ¶item 29 dropped, §item 26 dropped

Supplementary Table 4: VILL-33 item fit statistics

Item	Infit mean-square	Outfit mean-square
**Reading and accessing information subscale**		
Adjusting to the dark when entering a dimly lit room? (e.g. a restaurant at night)	1.13	1.04
Recognizing small objects in dim lighting? (e.g. coins)	1.12	1.13
Recognizing people's faces outside during dusk?	1.07	1.10
Recognizing people or objects by candlelight?	1.03	1.12

Terheyden JH, *et al. Br J Ophthalmol* 2023; 107:1144–1150. doi: 10.1136/bjophthalmol-2021-320848

Seeing things clearly close up in the middle of your field of vision?	1.32	1.33
Reading print which has a low contrast to its background?	0.79	0.80
Reading print which is not black? (e.g. grey)	0.81	0.81
Reading text on a digital display? (e.g. in the car, on an electronic radio)	1.23	1.35
Reading print against a colourful background? (e.g. a brochure)	1.07	1.01
Reading a paperback novel in dim lighting?	0.65	0.63
Reading a newspaper in dim lighting?	0.66	0.64
Reading a menu in a dimly lit restaurant?	0.79	0.74
Reading labels or instructions on medicine bottles in good lighting?	1.32	1.24
Reading labels or instructions on medicine bottles in dim lighting?	0.81	0.81
Reading package labels or price tags in a shop?	1.04	1.01
Felt exhausted by reading in dim light?	1.18	1.18
Needed additional lighting to see or read anything?	1.26	1.19
**Mobility and safety subscale**		
Driving a car on a sunny day? (with or without sunglasses)	1.22	1.23
Driving a car along a road lined with trees on a sunny day? (with or without sunglasses)	1.03	0.96
Driving a car at night?	0.84	0.80
Driving a car at night in the rain?	0.80	0.80
Reading street signs in time when driving by?	1.29	1.26
Walking on uneven ground in the dark?	0.92	0.97
Going out to do things during dusk? (e.g. visiting the supermarket or shops)	1.04	0.99
Seeing steps or curbs in the dark?	0.76	0.81
Getting your bearings in dimly lit or dark unfamiliar places?	0.80	0.83
Felt blinded by oncoming cars at night?	1.11	1.08
Felt blinded by the sun whilst driving a car? (with or without sunglasses)	1.09	1.09
Felt unsafe as a pedestrian or cyclist at dawn or at night?	1.40	1.13
**Emotional well-being subscale**		
Felt worried that your eyesight might get worse?	1.35	1.38
Felt worried about losing your independence?	0.87	0.81
Felt worried about the future?	0.86	0.83
Felt worried that your lifestyle might change due to your eye condition?	0.84	0.82
